# Characterization of the Genomic Diversity of Norovirus in Linked Patients Using a Metagenomic Deep Sequencing Approach

**DOI:** 10.3389/fmicb.2017.00073

**Published:** 2017-01-31

**Authors:** Neda Nasheri, Nicholas Petronella, Jennifer Ronholm, Sabah Bidawid, Nathalie Corneau

**Affiliations:** ^1^National Food Virology Reference Centre, Bureau of Microbial Hazards, Food Directorate, Health CanadaOttawa, ON, Canada; ^2^Biostatistics and Modeling Division, Bureau of Food Surveillance and Science Integration, Food Directorate, Health CanadaOttawa, ON, Canada; ^3^Department of Food Science and Agricultural Chemistry, Faculty of Agricultural and Environmental Sciences, Macdonald Campus, McGill UniversityMontreal, QC, Canada; ^4^Department of Animal Science, Faculty of Agricultural and Environmental Sciences, Macdonald Campus, McGill UniversityMontreal, QC, Canada

**Keywords:** norovirus, next-generation sequencing (NGS), linked patients, genetic variation, antigenic variation, recombination

## Abstract

Norovirus (NoV) is the leading cause of gastroenteritis worldwide. A robust cell culture system does not exist for NoV and therefore detailed characterization of outbreak and sporadic strains relies on molecular techniques. In this study, we employed a metagenomic approach that uses non-specific amplification followed by next-generation sequencing to whole genome sequence NoV genomes directly from clinical samples obtained from 8 linked patients. Enough sequencing depth was obtained for each sample to use a *de novo* assembly of near-complete genome sequences. The resultant consensus sequences were then used to identify inter-host nucleotide variations that occur after direct transmission, analyze amino acid variations in the major capsid protein, and provide evidence of recombination events. The analysis of intra-host quasispecies diversity was possible due to high coverage-depth. We also observed a linear relationship between NoV viral load in the clinical sample and the number of sequence reads that could be attributed to NoV. The method demonstrated here has the potential for future use in whole genome sequence analyses of other RNA viruses isolated from clinical, environmental, and food specimens.

## Introduction

Human norovirus (NoV) belongs to *Caliciviridae* family and its genome is comprised of a single stranded, positive sense RNA that contains 3 open reading frames (ORFs) coding for 9 non-structural and structural proteins (Green, 2013). NoV is the most frequent cause of infectious gastroenteritis worldwide (Scallan et al., 2011; Havelaar et al., 2015). While NoV infections are generally acute and self-limiting, persistent infections have been reported in immunocompromised and elderly patients (Aoki et al., 2010; Belliot et al., 2014; Green, 2014).

Based on genetic diversity in the capsid region NoVs are divided into 6 genogroups (GI–GVI) which are further divided into 36 genotypes (Kroneman et al., 2011; Vinje, 2015; Sarvestani et al., 2016). Nine genotypes of GI NoVs, 22 genotypes of GII, and a single genotype of GIV and GVI are known to affect humans (Ramirez et al., 2008; Bull and White, 2011; Moore et al., 2015). Genogroup II is the most prevalent genogroup, which accounts for 96% of all infections, and GII.4 is the most common genotype globally (Bull and White, 2011; Eden et al., 2013; Vega et al., 2014). NoVs are known to undergo rapid evolution. The overall mutation rate for the viral capsid protein, which determines the antigenic profile, is 4.16 × 10^−3^ nucleotide substitutions per site per year and this rate is consistent with other RNA viruses (10^−3^–10^−5^ per site per year). The mutation rate is slightly higher for GII.4 (4.3 × 10^−3^ per site per year), and this is believed to provide the GII.4 strains with higher epidemiological fitness (Bull et al., 2010; Boon et al., 2011). There is evidence that GII.17 is replacing GII.4 as the predominant genotype in certain parts of the world, but detailed epidemiological studies have yet to be conducted to determine the mutation rate of GII.17 (Chan et al., 2015; Chen et al., 2015; de Graaf et al., 2015; Parra and Green, 2015). Genetic diversity also has fitness benefits for NoV, such as increased viral replication, antigenic variability, and enhanced virulence. Antigenic variability is particularly important since antigenic epitopes are under constant evolutionary pressure from the host immune system and novel strain emergence often results in pandemic NoV outbreaks (Karst and Baric, 2015; de Graaf et al., 2016). Therefore, the ability to identify intra- and inter-host NoV genetic diversity and evolutionary hotspots could lead to a better understanding of the processes underlying viral evolution and novel strain emergence, and this has the potential to aid in vaccine development.

NoV can be spread through multiple routes; although, person-to-person and foodborne transmission are the most common (Hall et al., 2013; Barclay et al., 2014; Verhoef et al., 2015). Due to lack of surveillance data, attribution of NoV cases or outbreaks to specific food commodities, as well as determining the direction of NoV transmission after person-to-person spread is often challenging (Barclay et al., 2014; Moore et al., 2015). An effective approach to controlling NoV infection is to understand the mechanism and direction of transmission and then to enact an effective intervention strategy.

Whole genome sequencing (WGS) data can be used to elucidate phylogenetic relationships, monitor the transmission chains and evolution of viruses. For example, during the recent (2014–2016) Ebola outbreak, a WGS approach was adopted to reveal valuable information regarding the viral transmission dynamics, genomic variations (Gire et al., 2014), and allowed for identification of new signatures for prediction of emerging strains (Sozhamannan et al., 2015). Post-outbreak, retrospective WGS analysis can also be performed on NoV strains to examine quasispecies evolution and predict the development of emerging strains through identification of rare variants with predictable signatures that could render predominance. Therefore, WGS analysis of NoV has the potential to provide fast and accurate source attribution, tracking of transmission direction, and prediction of emerging strains (Kundu et al., 2013; Barclay et al., 2014; Karst and Baric, 2015).

Next-generation sequencing (NGS) approaches now offer routine WGS in research and testing laboratories; however, NGS protocols have yet to be developed for all potential applications (Ronholm et al., 2016). WGS of NoV, directly from clinical samples is routinely accomplished by performing RT-PCR to amplify viral RNA using several sequence-specific and overlapping primer sets (Kundu et al., 2013; Cotten et al., 2014). However, the use of sequence-specific primer sets introduces amplification bias and the assembly of amplicons into WGS assumes conserved viral synteny, this result in overlooking genomic variations. Also, generating whole genome sequences using this approach for even small number of samples is laborious (Thomson et al., 2016). Recently a target enrichment library preparation approach based on SureSelect sequence-specific probes was successfully employed for WGS analysis of NoVs from clinical samples (Brown et al., 2016). While this approach overcomes the problem of primer design in amplicon sequencing, it is costly, requires the prior knowledge of genotype, and is only more effective compared to conventional RNA-Seq approach for samples with low viral loads (Thomson et al., 2016). In this study, we have employed a rapid and unbiased metagenomic NGS approach for NoV from clinical samples, which also provides enough coverage depth for identification of minor variants within viral quasispecies (Mancuso et al., 2011; Iles et al., 2015). Bias was further reduced by using *de novo* assembly. In each case sequencing coverage was sufficient for assembly into a single contiguous sequence. We have also performed a comprehensive genomic analysis during direct transmission of NoV from one patient to another by investigating NoV infection in linked patients. We analyzed the data to provide a detailed description of the viral genome and the presence of inter- and intra-host variants, as well as genome-wide recombination events.

## Materials and methods

### Sample collection and preparation

Fecal samples from families in Ottawa, Ontario who reported symptoms of diarrhea and vomiting were submitted to the National Food Virology Reference Centre at Health Canada for verification of NoV infection. We chose 8 NoV positive samples from 4 families submitted 2013–2015 for our study (Table 1). In all cases the source of original transmission was most likely person-to-person and the direction of transmission was known through the recorded epidemiological data. In family A patient 13–38 infected 13–39, in family B 14–55 infected 14–56, in family C14–58 infected 14–59, and in family D15–65 infected 15–66. In each transmission event the infectious source was a toddler, and the secondary infection was a parent for families A, B, and D, and a sibling for family C.

**Table 1:** ***De novo* assembly of NoV genomes from clinical samples obtained from linked patients**.

**Family group**	**Sample ID**	**Genotype**	**Viral Load**	**Total no. Reads**	**Total Viral Reads**	**% Viral Reads**	**Fold Coverage**
A	13–38	GII.4	17640	12,042,724	7163	0.06	976
A	13–39	GII.4	23606	4,301,150	12,373	0.29	121
B	14–55	GII.6	60400	3,950,778	22,326	0.57	223
B	14–56	GII.6	9814	12,493,937	7939	0.06	121.5
C	15–58	GII.4	20159	2,730,201	8766	0.32	75.5
C	15–59	GII.4	61600	3,473,043	29,427	0.85	261
D	15–65	GII.6	6268	5,406,137	2946	0.05	309.5
D	15–66^*^	GII.6	2706	12,156,220	566	0.00	72

*Viral titre is calculated as genome copies/μl*.

* *Consensus sequence obtained from 15–65 was used as a reference for the assembly of 15–66*.

Ten percent stool suspensions were clarified by centrifugation (6000 × g for 5 min) and the supernatant was filtered through a 0.45 μM then 0.22 μM filter (Millipore, Etobicoke, Ontario, Canada). RNA was extracted from filtrate using the Viral RNA Mini Kit (Qiagen, Mississauga, Ontario, Canada) according to the manufacturer's protocol. Reverse transcription (RT)-PCR was conducted using a One-Step RT-PCR kit (Qiagen) according to the manufacturer's instructions and primers as described previously (Mattison et al., 2010). Following electrophoresis and in-gel visualization, the amplified products were extracted with the Qiagen gel-purification kit and were sequenced with an ABI3130 Genetic Analyzer to validate NoV infection and for genotype determination.

Viral titres were determined by droplet digital PCR (Bio-Rad, Hercules, California, USA) (Racki et al., 2014) using the probes and primers that were described previously (Kageyama et al., 2003). For this purpose, 20 μL of each reaction mix was converted to droplets with the QX200 droplet generator (Bio-Rad). Droplet-partitioned samples were then be transferred to a 96-well plate, sealed and cycled in a C1000 deep well Thermocycler (Bio-Rad) under the following cycling protocol: 42°C for 30 min (RT) and 95°C for 5 min (DNA polymerase activation), followed by 45 cycles of 95°C for 30 s (denaturation) and 50°C for 1 min (annealing) followed by post-cycling steps of 98°C for 10 min (enzyme inactivation), and then an infinite 4°C hold. The cycled plate was transferred and read in the FAM and HEX channels using the QX200 reader (Bio-Rad).

### RNA-Seq library preparation

The quality and quantity of extracted RNA was examined using Agilent RNA 6000 Pico Assay Kit and Protocol (Agilent Technologies, Santa Clara, California, USA). Ethanol precipitation of RNA was performed prior to proceeding to TruSeq Stranded mRNA (Illumina, San Deigo, California, USA) sample preparation according to the manufacturer's instructions. Briefly, RNA pellets from ethanol precipitates were suspended in Elute, Prime, and Fragment Mix solution, followed by tagmentation at 94°C for 8 min. The first strand cDNA synthesis was conducted using SuperScript III (ThermoFisher Scientific, Waltham, Massachusetts, USA) and random hexamer primers at 25°C for 10 min, 50°C for 50 min, 70°C for 15 min. Second Strand Master Mix was added into the original reaction mixture and used to synthesize the second cDNA strand at 16°C for 60 min. The Agencourt AMPure XP—PCR Purification system (Beckman Coulter Canada, Mississauga, Ontario, Canada) was used to purify the complete double-stranded cDNA. Next A-Tailing Mix was added to the purified cDNA by incubating at 37°C for 30 min to adenylate the 3′ ends. Ligation to commercial adapters, a thymidine overhang from the indexed paired-ends adapters, was conducted at 30°C for 10 min by adding DNA Ligase Mix and adapters to the sample DNA. To stop the ligation, Stop Ligase Buffer was added into the reaction mixture. The Agencourt AMPure XP—PCR Purification system (Beckman Coulter Canada) was used to purify adapter-ligated-DNA products. Library enrichment was performed by adding a PCR MasterMix at 98°C for 30 s and 16 cycles of 98°C for 10 s, 60°C for 30 s, and 72°C for 30 s, with a final extension at 72°C for 5 min. The enriched library was purified by the Agencourt AMPure XP—PCR Purification system (Beckman Coulter Canada), followed by elution with Resuspension Buffer. DNA quantity and quality was measured using Agilent High Sensitivity DNA Assay (Agilent Technologies) as well as the Quant-iT High-Sensitivity dsDNA Assay (ThermoFisher Scientific). The sample preparations were pooled at a concentration of 1 nM of DNA. Freshly diluted NaOH (0.2N) was added to equal volumes of the DNA libraries for denaturation, followed by further dilution with pre-chilled HT1 buffer to obtain a final concentration of 8 pM in a total volume of 1 ml. A 1% final PhiX concentration was added to the denatured DNA for use as an internal control. The prepared DNA library was loaded into a 150 cycle MiSeq Reagent Kit v3 and paired-end sequenced for 76 bp in each direction. The data demonstrated in this article are obtained from the sum of two MiSeq runs. The yield for the first run was 4.52 Gbp (35.1 M reads) and for the second run was 4.78 Gbp (41.3 M reads).

### Sequence assembly and analysis

The raw sequence reads for each sample consisted primarily of non-NoV reads; therefore, preliminary computational steps needed to be used to ensure an adequate NoV assembly. An in-house database was created that comprised all of the NoV sequences available from NCBI. This database included both complete and partial NoV genomes as well as all available complete and partial open reading frames and coding sequence (CDS) genes. The raw sequence reads for each sample were aligned, using the BLASTn algorithm, to the in-house NoV database, all reads that matched a database entry were binned by the corresponding hit's GenInfo Identifier (GI). Each bin was inspected to identify which NoV database sequences were hit, how many sequence reads were associated with a given GI number, and which complete NoV genome entry had the most hits from a sample. To produce a *de novo* whole genome assembly from sequence reads matching the NoV database bins were concatenated and concatenated reads were assembled into contigs using SPAdes (Bankevich et al., 2012).

Additionally, the previously identified closed genome with the highest number of associated reads was then used in a reference guided assembly using SMALT v 0.7.4 (https://sourceforge.net/projects/smalt/). SMALT was chosen because we have already observed that it performed well with low coverages and when using distant references (Pightling et al., 2014). This was performed to identify variants to the reference genome. Variants were reported using Bcftools (Li, 2011).

The custom script and database used to perform assemblies is available at https://sourceforge.net/projects/norobin. The SRA accession numbers of each *de novo* assembly are as follows: SRR3441741, SRR3458065, SRR3458066, SRR3458067, SRR3458068, SRR3458069, SRR3458070, SRR3458071. Throughout this article, we refer to the variations within the viral quasispecies as single nucleotide variations (SNV), and the variations between the consensus sequences obtained from *de novo* assemblies as single nucleotide polymorphisms (SNP).

### Phylogenetic analysis

The NoV consensus sequences obtained from each *de novo* assembly were aligned with chosen reference genomes obtained from GenBank using MUSCLE (Edgar, 2004). The phylogenetic trees were constructed from this alignment with RAxML implementing a GTR Gamma nucleotide substitution model (Stamatakis, 2014) using the sequences from ORF1 and ORF2.

### Recombination analysis

Potential recombination within the complete genome sequences was screened using seven methods (RDP, GENECONV, MaxChi, Bootscan, Chimera, SiScan, and 3Seq) implemented in the Recombination Detection Program version 4.46 (RDP4) (Martin et al., 2015). The breakpoints were also defined by RDP4. Similarity between the recombinants and their possible major and minor parents was estimated using BootScan, embedded in RDP4 (Stamatakis, 2014). SimPlot (Lole et al., 1999) was used to visualize the relationships among the recombinants and their possible parents. The recombination event evaluated by RDP4 was considered significant if it satisfied at least 2 criteria when the *P* < 0.05 and the RDP recombination consensus score (RDPRCS) was >0.6^31^ (Kim et al., 2016).

## Results

### *De novo* assembly of norovirus sequences

We obtained 8 fecal samples from 8 symptomatic NoV patients belonging to 4 families (A–D). All samples were RT-PCR positive for NoV GII and further analyses demonstrated that four samples belong to GII.4 genotype (from families A and C) and four samples belong to GII.6 genotype (from families B and D) (Table 1).

Near-full-length genome sequences (78.9–99.9% coverage length) were generated from all 8 samples (GenBank accession numbers: KX158279, KX158280, KX158281, KX158282, KX158283, KX158284, KX158285, KX158286). The total number of sequence reads from each sample varied between 2.7 and 12.5 million (Table 1) and the proportion of reads that matched a NoV reference sequence also varied between 0.005 and 0.85% of the total sequence reads. This observation is consistent with previous reports for other RNA viruses (Wong et al., 2013). Average coverage depth across genomes was 72- to 976-fold with an average of 270-fold (Table 1). It appears that the minimum number of sequence reads required to obtain an accurate full-length consensus sequence for norovirus was 2900 (Table 1). There was not a correlation between total number of reads and percentage of viral reads (*r* = −0.565). Seven out of 8 sequenced samples produced sufficient reads to allow 90% of the reference genome bases to be called. However, sample 15–66 yielded a low number of reads therefore we used the 15–65 sequence as a reference for the assembly of 15–66.

### The relation between viral titre and depth of coverage

**Figure 1:**
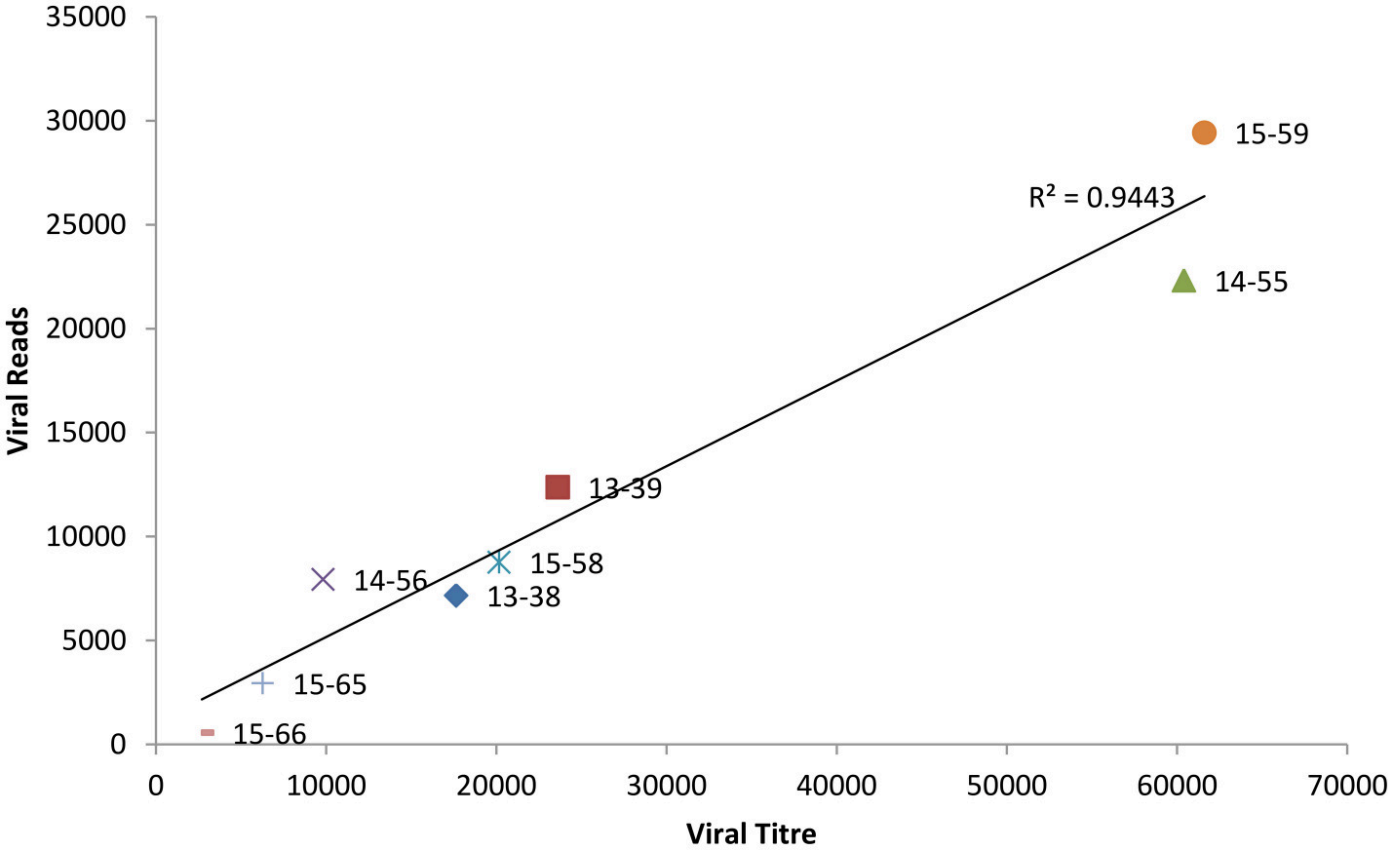
**Correlation between the viral titre (genome copies/μl) and depth of coverage (reads mapped to norovirus genome)**.

Whole genome sequence analyses of other RNA viruses have demonstrated that the depth of coverage improves by increasing viral load (Wong et al., 2013; Logan et al., 2014). To elucidate if the correlation between NoV viral titre and the depth of coverage was linear, we plotted the total number of viral reads versus viral titre (genome copies/μl). As shown in Figure 1, we observed a strong correlation (*r* = 0.972) between viral titer and depth of coverage. Samples with highest viral load exhibited higher coverage, while samples with lower titre yield low coverage.

### Distribution of viral reads

**Figure 2:**
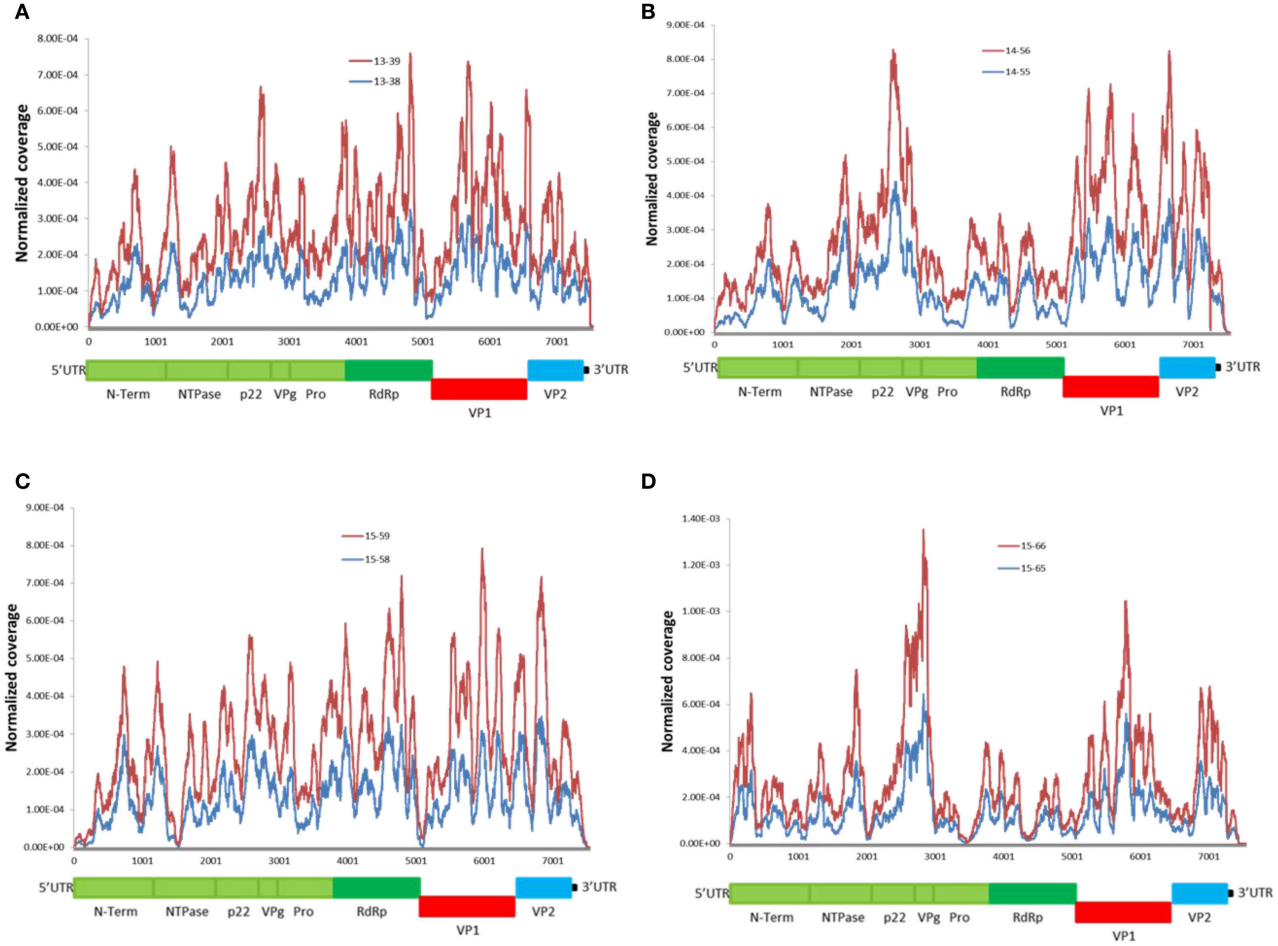
**Distribution of NoV reads across the sequenced genomes**. Coverage was calculated as the total number of reads covering a given nucleotide and was normalized by the sum of total coverage across the genome. i.e., at each residue, the coverage was divided by the total coverage and the sum of normalized coverage equals one. **(A–D)** read coverage for the linked patients from families **(A–D)** (Table 1). Schematic representation of the NoV encoded proteins is shown below the graphs

We examined genome-wide depth of coverage for each of the genomes by measuring the number of reads per position. The coverage across the length of the genome was not even. Sequence coverage was the highest in the mid ORF1 and ORF2 regions, and lower at the 5′and 3′ ends of genomes (Figure 2). This finding is consistent with other studies reporting difficulty in recovering readable fragments from Illumina short-read sequences at the end of DNA molecules (Mortazavi et al., 2008; Batty et al., 2013). The overall coverage profile was consistent between the linked genomes (Figure 2), as well as between genomes from the same genotype (Supplementary Figure 1). This suggests that the depth of coverage can be affected by an intrinsic property of the viral RNA genome. All sequenced genomes had low coverage depth at the intersection of ORF1-ORF2 (Figure 2). This could be explained by the presence of highly conserved and functional structured region at the junction of ORF1-ORF2 (Simmonds et al., 2008; Alhatlani et al., 2015). There is evidence that the 5′-end of ORF2 and the subgenomic RNA (sgRNA) contains extensive RNA secondary structures that function as the promoter for ORF2. Disruption of these evolutionarily conserved RNA stem-loops severely decreases viral replication (Simmonds et al., 2008; Alhatlani et al., 2015). Therefore, our observations further suggest that RNA secondary structures appear to interfere with coverage depth.

### Phylogenetic analyses

**Figure 3:**
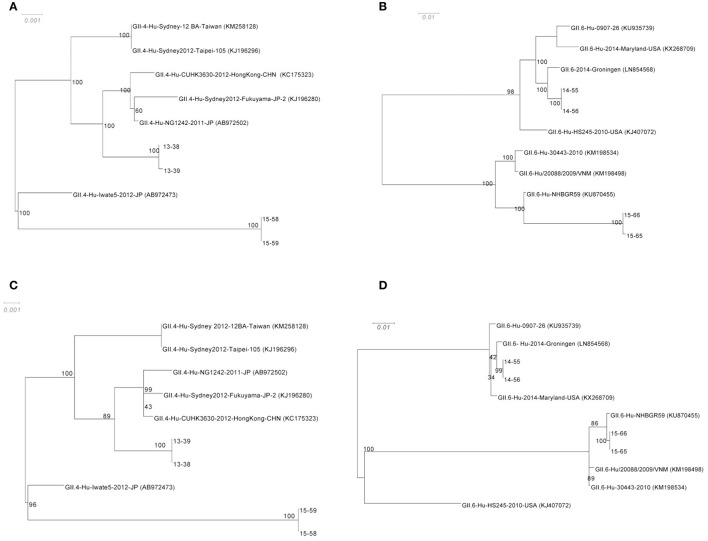
**Phylogenetic analysis of the NoV genomes identified in this study**. The consensus sequences of the ORF1 for GII.4 **(A)** and GII.6 **(B)** sequences respectively, and ORF2 for GII.4 **(C)** and GII.6 **(D)** from each assembly were then aligned with highly similar sequences from GenBank database. The robustness of the phylogeny was assessed through bootstrap analysis of 1000 pseudo-replicates. The accession numbers for the reference genomes are provided in parentheses. The scale bars represent the number of substitutions per site.

Phylogenetic trees were constructed from the alignment of the censuses sequences obtained from *de novo* assembly of complete ORF1 (Figures 3A,B), and complete ORF 2 (Figures 3C,D) with highly similar sequences from the NCBI database. To assess the robustness of each node, a bootstrap resampling process was performed (1000 replicates) and demonstrated at each node. As shown, the phylogenetic relationships between GII.4 sequences (13–38, and 13–39, as well as 15–58, and 15–59, from families A and C respectively) and their closest relatives remain similar for both ORF1 and ORF2 (Figures 3A,C). Sequences obtained from 13–38, and 13–39 cluster with GII.4-Hu-CUHK3630-2012-HongKong-CHN (Accession No: KC175323), GII.4-Hu-Sydney2012-Fukuyama-JP-2 (Accession No: KJ196280), and GII.4-Hu-NG1242-2011-JP (Accession No: AB972502). Sequences obtained from 15–58 ad 15–59 show homology to GII.4-Hu-Iwate5-2012-JP (Accession No: AB972473) at both ORFs. However, the phylogenetic relationships between GII.6 sequences (14–55, 14–56 from family B and 15–65, 15–66 from family D) and their closest relatives differ slightly between ORF1 and ORF2 (Figures 3B,D). It appears that there is more distance between 15–65 and 15–66 sequences and their reference (GII.6-Hu-NHBGR59, Accession No: KU870455) at ORF1 compared to ORF2, as there are 231 SNPs between these genomes and their reference at ORF1, while only 61 SNPs were detected at ORF2 (Figures 3A,B). Sequences obtained from 14–55 and 14–56 showed homology to GII.6/2014/Groningen (Accession No: LN854568).

### Investigation of SNVs across the NoV genome

Multiple lines of evidence suggest that the diversity of the NoV quasispecies in infected individuals with a normal immune system is quite low (Bull et al., 2012; Karst and Baric, 2015). We observed a high similarity between the consensus sequences obtained from linked patients. However, there were several SNPs between the linked genomes that are listed in Table 2. While the consensus sequences obtained from family C (15–58/15–59) showed 100% similarity, the consensus sequences gained from family A (13–38/13–39), family B (14–55/14–56), and family D (15–65/15–66) demonstrated 2, 1, and 4 SNPs across genome, respectively (Table 2).

**Table 2:** **Single nucleotide polymorphisms between the linked genomes**.

**Family**	**Linked genomes**	Position^*^	**Region**	**Variation**
A	13–38/13–39	829	ORF1	G/A
		7447	ORF3	C/T
B	14–55/14–56	3133	ORF1	G/A
C	15–58/15–59	–	–	None
D	15–65/15–66	346	ORF1	G/A
		1102	ORF1	C/T
		1114	ORF1	A/G
		3484	ORF1	C/T

* *Nucleotides are numbered according to the NoV GII4/2012/Sydney (Accession No: KM258128)*.

**Figure 4:**
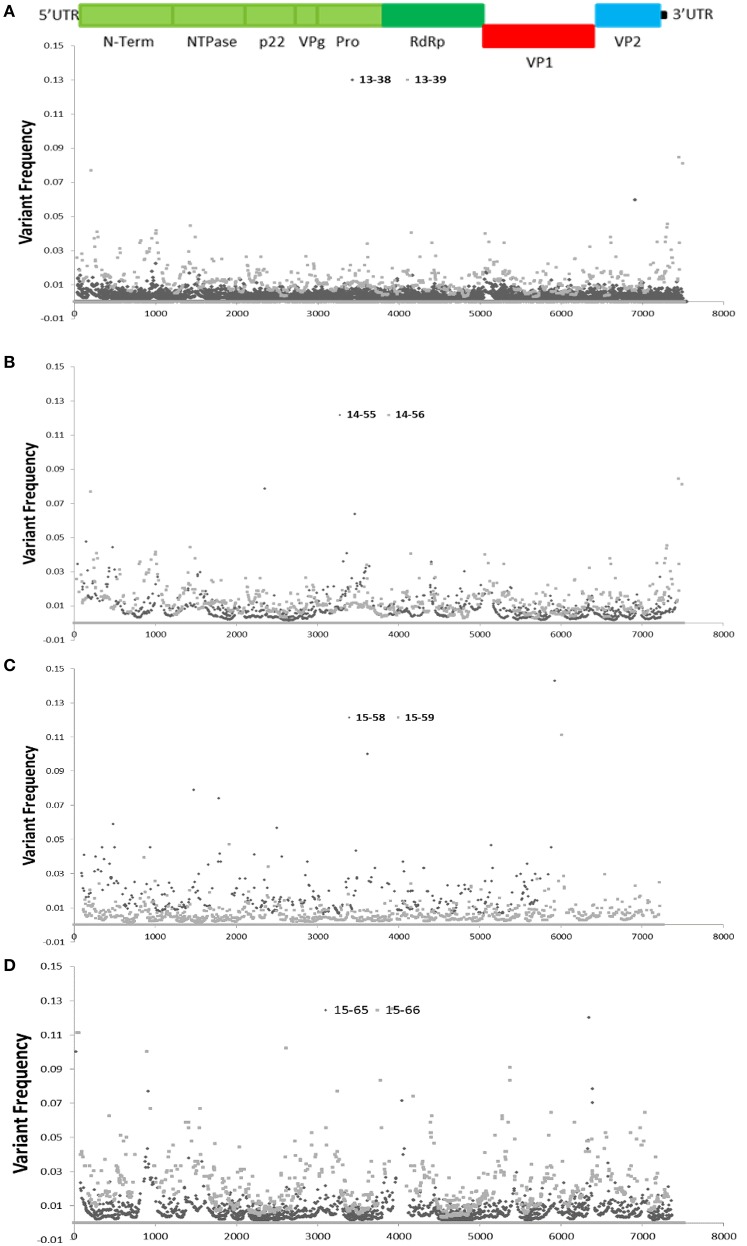
**Distribution of single nucleotide variants (SNVs) across the sequenced NoV genomes**. Schematic representation of the NoV encoded proteins is shown above the graphs. **(A–D)**, graphs represent SNV frequency at each position for families **(A–D)**.

High coverage depth facilitates identifying a variety of unique intra-host variants at different frequencies. The variability of nucleotides observed at each position of the sequenced NoV genomes (with coverage depth of 10-fold or higher) was assessed by analysis of sequence variations at each genome position, and demonstrated in dot plots for each family (Figure 4). Since the anticipated sequence error rate for Illumina is approximately 2% (Erik Garrison, 2012; Hasing et al., 2016), genetic variations that occur at frequencies higher than 2% can potentially be natural SNVs. As demonstrated in Figure 4, the areas with higher variability are observed at the RdRP, VP1, and the VP2 (ORF3) regions with variant frequency of 5% or higher (Figure 4).

When mutations occur in the VP1 (ORF 2) protein, it leads to antigenic variation and subsequent immune evasion (White, 2014). The VP1 protein has three main domains: an N-terminal domain (N), a highly conserved shell domain (S), and a protruding domain (P) which forms surface-exposed spikes on the virus surface. The P domain is further divided into hypervariable domain (P2) and a more conserved P1 domain (P1) (Shanker et al., 2011; Debbink et al., 2012). Within the P2 subdomain 5 highly variable blockade epitopes have been defined (A–E). These epitopes surround the histoblood group antigen (HBGA) binding pocket, Epitope A is considered to be the most important determinant of antigenic variation (Lindesmith et al., 2013). However, other residues adjacent to these epitopes might also impact NoV affinity to HBGAs and binding to neutralizing antibodies (Giammanco et al., 2014; White, 2014).

**Figure 5:**
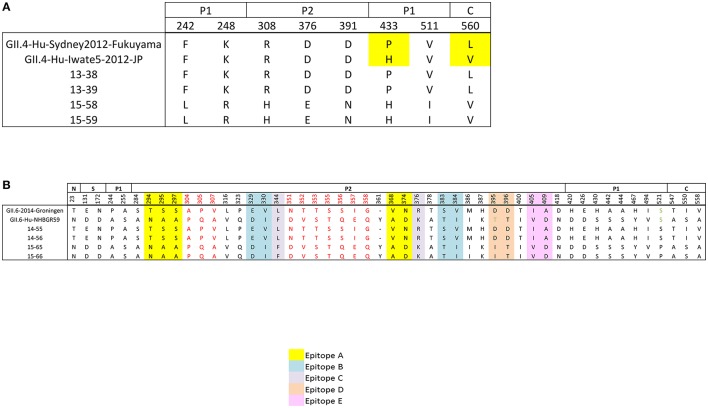
**Non-synonymous differences within the structural domains of the capsid protein (VP1, ORF2), which are the N-terminal (N), shell (S), P1, and P2 domains. (A)** Non-synonymous differences between the sequenced GII.4 Sydney genomes (families A and C) and their references **(B)** amino acid differences between the sequenced GII.6 genomes (families B and D) and their references. Individual epitope sites are highlighted in different colors: Yellow, epitope site A; blue, epitope site B; gray, epitope site C; orange, epitope site D; pink, epitope site E. Stretches of amino acid differences are shown in red. Residues are numbered according to the NoV GII4/2012/Sydney (Accession No: KM258128).

To study the variation within the VP1 protein between the patients infected with the same genotype, we performed amino acid sequence alignment of VP1 protein (Figures 5A,B). Amino acid sequence alignment of GII.4 Sydney-2012 samples demonstrated no coding change in the conserved N and S domains, 4 in the P1 domain, and 3 in the P2 domain with D376E falling in the C epitope and D391N in the D epitope. Also one non-synonymous change was detected in the C domain (Figure 5A).

Unlike the GII.4 Sydney-2012 isolates, the GII.6 isolates do not belong to the same strain (Figure 3). Therefore, higher amino acid sequence variability is expected in the complete VP1 protein of GII.6 isolates (Figure 5B). The number of non-synonymous differences between samples 14–55, 14–56, and 15–65, 15–66 was 1, 2, 10, 34, and 3 for N, S, P1, P2, and C domains, respectively. Each blockade epitope had non-synonymous SNPs, with 5 differences in epitope A (T294N, S295A, S297A, V368A, N374D). These differences may have a marked effect on the antigenicity, since it has been previously shown that residues 294 and 368 play key roles in the blockade responses (Vongpunsawad et al., 2013). Two stretches of coding differences that are not within the putative epitopes: one A304P, P305Q, V307A and a relatively longer stretch N351D, T352V, T353S, S355T, S356Q, I357E, G358Q (Figure 5B) were observed. While most of these coding differences exist in the reference genomes, there are several unique coding changes that are only present in the genomes sequenced in this study such as D395I (15–65 and 15–66) and S521P (14–55 and 14–56). Collectively, these data suggest that strains belonging to the same genotype can be antigenetically different, and this supports the hypothesis that the emergence of new strains within a certain genotype is the result of escape from herd immunity as they undergo evolution in antigenic and surface-exposing residues.

### Recombination analyses

Recombination is a significant source of genetic diversity in NoVs. This phenomenon, which occurs during co-infection with different strains, is usually observed at the ORF1-ORF2 and ORF2-ORF3 junctions (White, 2014). There is growing evidence that the recombination rate is increasing among noroviruses (Bull et al., 2012; Wong et al., 2013; Lim et al., 2016); however, the lack of sufficient full-length NoV sequence data has hindered the search for inter- and intra-genotype recombination, and has limited the understanding of how recombination affects NoV evolution (Eden et al., 2013). Therefore, in this study we investigated the incidence of recombination throughout the NoV genomes. Through phylogenetic analysis and separate genotyping of ORF1, and ORF2 regions using Noronet genotyping tool (Kroneman et al., 2011), it was found that all GII.4 Sydney 2012 genomes possessed the original GII.Pe ORF1 (Martella et al., 2013, Table 3) and all GII.6 genomes were associated with GII.P7 ORF1, which is a predominant recombination variant of GII.6 strains (Fumian et al., 2016; Lim et al., 2016).

**Table 3:** **Recombination analysis using Noronet genotyping tool**.

**Sample ID**	**Length**	**ORF1 genotype**	**ORF2 genotype**	**ORF1 genotype support**	**ORF2 genotype support**
13–38	7542	GII.Pe	GII.4 Sydney_2012	100	100
13–39	7512	GII.Pe	GII.4 Sydney_2012	100	100
15–58	5963	GII.Pe	GII.4 Sydney_2012	100	100
15–59	7356	GII.Pe	GII.4 Sydney_2012	100	100
15–55	7539	GII.P7	GII.6	100	100
14–56	7491	GII.P7	GII.6	100	100
15–65	7586	GII.P7	GII.6	100	100
15–66	7330	GII.P7	GII.6	100	100

**Figure 6:**
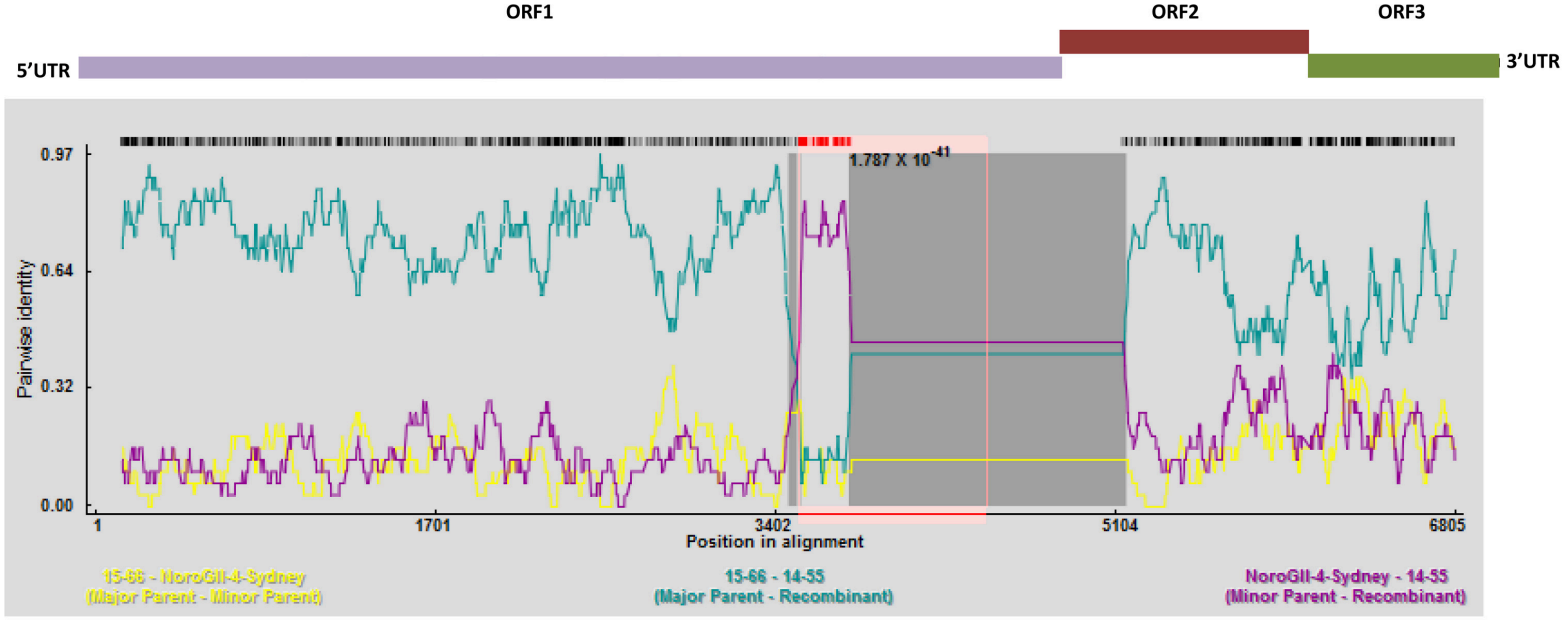
**Recombination analyses of the full genomes of norovirus**. A sliding window of 200 nt was utilized to make the SimPlot. SimPlot was generated for the linked recombinant genomes 15–65 and 15–66. Two breakpoints were identified, which leads to production of a mosaic genome that has a GII.6 backbone with an insertion from a GII.4 Sydney. Schematic representation of the NoV genome is shown above the graph.

To perform an in depth analyses of recombination, a genome-wide examination of sequences from the same genotype using RDP4 was preformed (Figure 6). This program analyses sequences for the presence of recombination using seven different recombination detection methods (RDP, GeneConv, Bootscan, MaxChi, Chimaera, SiScan, and 3Seq), and provides the statistical probability of each recombination event (Martin et al., 2015). As shown in Figure 6, there is evidence of a major recombination event between the 15–65, 15–66, and GII.4 Sydney (Accession No: KM258128) genomes at nucleotide positions 3527–5163, which encompasses the 3′ end of the ORF1. This recombination event was unique to 15–65, 15–66 genomes and was not detected in 14–55 and 14–56 genomes. Moreover, further recombination analysis by RDP4 demonstrated that all the GII.4 Sydney genomes (13–38, 13–39, 15–58, and 15–59) contained the same polymerase (GII.Pe), which is considered a signature of the pandemic Sydney 2012 strains (Martella et al., 2013) (data not shown).

## Discussion

Noroviruses are genetically diverse and this heterogeneity increases the adaptability and fitness of certain variants. Novel antigenic variants can emerge and escape herd immunity or strains with increased replication rate or environmental persistence can appear that spread more rapidly and efficiently than others. NoVs genetic diversity is influenced by viral factors such as polymerase errors, genetic recombination, and host factors, such as the immune response. It has been hypothesized that inter- and intra-host genetic heterogeneity can act as a reservoir for new variants. Since NoV, like many other RNA viruses, exists as a swarm of quasispecies, it is not clear how much genetic variation can be expected during direct transmission of an acute infection. Therefore, the virus that is isolated from the source of transmission might not necessarily be genetically identical to the virus isolated from the immediate patient. Knowing the range of variation possible between each transmission event is valuable, and our study examined the genetic diversity between a primary and a secondary infection during a single person-to-person transmission events. This is the first step toward facilitating more accurate source attribution as well as establishing a method for determining the directionally of transmission during outbreaks.

Noroviruses generally cause acute and self-limiting infections, therefore we aimed to examine the intra- and inter-patient genetic variations throughout the entire NoV genome isolated from symptomatic, acutely infected, linked patients. For that purpose, next-generation sequencing was used to analyze whole NoV genomes obtained from clinical samples of 8 linked patients (4 GII.4 and 4 GII.6) belonging to 4 families. We selected NoV GII.4 for examination in this investigation because it is the predominant genotype in the North America (de Graaf et al., 2015, 2016), and GII.6 since it is the second most prevalent genotype (Chan-It et al., 2012; Vega et al., 2014; Luo et al., 2015; Xue et al., 2015).

To design a WGS strategy that was unbiased by sequence-specific primers, we employed a metagenomic approach for RNA-Seq on Illumina MiSeq platform to sequence NoV genomes directly from clinical samples. By selecting only sequence-reads that aligned to known NoV sequences, *de novo* assembly of the entire NoV genome was possible (Table 1). High coverage-depth made it possible to identify minor variants at each nucleotide position. We have shown that clinical samples contain sufficient viral RNA to perform successful virus WGS, despite the presence of sequence reads from other sources such as the host, dietary materials, and microbial RNA. Only one sample (15–66), which also had the lowest viral load, did not yield sufficient reads for *de novo* assembly and therefore we performed reference-guided assembly using its linked genome, 15–65, as the reference. Overall the bases which could not be sequenced were clustered at the ends of the genome, which consistently yields low coverage (Figure 2). Low coverage at the ends of the genome is consistent with other reports that the 5′ and 3′ terminals of viral RNA genomes are difficult to sequence (Batty et al., 2013). This is particularly evident in samples with low viral titre, suggesting that increasing the input RNA of such samples could improve coverage. We confirmed the correlation between depth of coverage and viral load by plotting coverage against the viral titre and calculating the Pearson correlation coefficient (Figure 1). However, in a study conducted on Measles virus, no correlation was found between the viral titre in samples and the fraction of the genome covered (Penedos et al., 2015).

Variability in coverage and sequencing depth across the genome was observed with all samples; however, overall coverage profile in the linked genomes, as well as genomes that belong to the same genotype, is strikingly similar (Figure 2 and Supplementary Figure 1). While the reason for this observation is not clear, we hypothesized that the presence of RNA secondary structures in the viral genome influences the coverage depth and since the linked genomes have very similar sequences (Table 2), it is assumed that their secondary and tertiary RNA structures are also similar, which yield similar coverage profile. This assumption was further supported by the observation that in all the sequenced genomes, the coverage decreases at the junction of ORF1 and ORF2, where a highly structured promoter for ORF2 exists (Figure 2, Simmonds et al., 2008; Alhatlani et al., 2015; Yunus et al., 2015). The negative effect of RNA secondary structure on depth of coverage produced by RNA-Seq has been recently reported by other researchers as well (Biswas and Gao, 2016). RNA structure can affect target accessibility and limit the efficiency of RNA tagmentation and cDNA synthesis. Therefore, a heat-denaturation step prior to RNA tagmentation and cDNA synthesis may improve coverage for WGS of RNA viruses. Moreover, the overall coverage profile obtained from our sample is consistent with what Batty and colleagues have formerly reported (Batty et al., 2013).

For the phylogenetic analysis, a comparison was made in the genetic distance between the genomes sequences assembled in this study and their highly similar relatives for both ORF1 and ORF2 (Figure 3). While the genetic distance for the GII.4 Sydney genomes (13–38, 13–39, 15–58, and 15–59) remained consistent for both ORF1 and ORF2, 15–65 and 15–66 sequences that belong to GII.6 appeared to be closer to their reference GII.6-Hu-NHBGR59 in ORF2 when compared to ORF1. This phenomenon can be explained by the presence of a recombination event at the end of ORF1 for these genomes (Figure 6).

As expected, there are limited numbers of nucleotide differences between the consensus sequences obtained from linked genomes (Table 2). Nevertheless, it is notable that the SNPs that are observed between the linked genomes are missing in the reference genomes (data not shown), this might indicate that these SNPs uniquely occurred during the single transmission events.

Due to high coverage-depth, a variety of unique intra-host SNVs throughout the sequenced genomes was identified. The anticipated sequencing error rate by Illumina technology is approximately 2% (Bravo and Irizarry, 2010; Beerenwinkel et al., 2012) therefore it is generally accepted that genetic variations with frequencies ≥ 2 % and ≥ 5X coverage can be considered as potential SNVs (Erik Garrison, 2012). Other groups have also employed similar approach to identify SNVs within viral quasispecies (Faison et al., 2014; Penedos et al., 2015; Hasing et al., 2016; King et al., 2016). In this study the SNV frequency has been only demonstrated for the positions with 10-fold or higher coverage. While there are low frequency variants that can be considered as sequencing or amplification errors, many of high frequency variants, for example those that clustered within ORF2, the 3′ end of ORF1 and ORF3 (Figure 4) can potentially be natural occurring intra-patient SNVs.

The distribution of SNVs indicates that intra-host viral populations were more homogenous for patients in families A and B, with only a few SNVs above 5%. In contrast, patients within families C and D presented a more heterogeneous intra-host population, as multiple SNVs with a frequency of 5% or higher in the viral population were distributed across the entire length of the genome. Overall, we observed that some regions of the NoV genomes are particularly prone to mutation (variant frequency > 5%). It has also been demonstrated that during direct viral transmissions minor variants within the donor viral quasispecies can present as major variants or consensus sequences in the recipient viral population (Kundu et al., 2013; Holzknecht et al., 2015). Therefore, epidemiological data together with deep WGS data obtained from linked cases and transmission chains can provide efficient means for source attribution and may help to identify signatures and patterns in the mutations throughout the viral genomes. In the future this may enable us to predict strain emergence, viral evolution, and allow us to intervene sooner in an outbreak setting.

Amino acid variations in the complete VP1 protein, including the P2 domain were analyzed. Interestingly there are several non-synonymous differences between the closely related GII.4 Sydney genomes (Figure 5A), with two amino acid changes falling within the antigenic epitope C and D. These changes can potentially alter the antigenic profile of these viruses and lead to escape from herd immunity. However, in-depth analyses are required to confirm this hypothesis. Amino acid variations were more common between the sequenced GII.6 genomes; specifically the P2 domain contained the most variability with 34 amino acid substitutions (Figure 5B). However, the N domain and S domain of the capsid protein were highly conserved. We also screened the antigenic epitope sites for the presence of non-synonymous changes and identified variations in all 5 blockade epitopes, including 5 differences in epitope A, 4 in epitope B, 2 in epitopes C, D, and E. These findings are particularly interesting as these epitopes have been shown to modulate HBGA binding and neutralization responses (Giammanco et al., 2014; White, 2014). Therefore, our data supports the hypothesis that immune evasion by antigenic variation at neutralizing epitopes is a driving mechanism behind new NoV strain emergence.

Most of the recombination events for noroviruses have been observed at the ORF1/2 overlap, which allow the exchange of nonstructural and structural genes, and therefore contribute to the emergence of new epidemic strains (Eden et al., 2013; de Graaf et al., 2016). Herein we also identified GII.P7/GII.6 recombinant viruses that are frequently reported and, unlike the other detected recombinant strains, have long period of circulation (since 2004) (Fumian et al., 2016; Lim et al., 2016). Moreover, we identified a novel inter-genotypic recombination event at the 3′ end of ORF 1 for 15–65 and 15–66 genomes (Figure 6), which led to the production of a mosaic genome with GII.6 backbone and GII.4 Sydney insertion. This observation is further validated by the phylogenies of the ORF 1 region presented in this study (Figures 3A,B). The acquisition of novel ORF1 regions has also been suggested to increase viral fitness that contribute to strain emergence, for example, by altering the replication rate or polymerase fidelity (Eden et al., 2013).

In conclusion, a metagenomic NGS approach can be employed to successfully sequence the whole genome of NoVs directly from clinical samples, without the need for sequence-specific enrichment. In this study, as proof-of-principle, we sequenced each sample to a very high-coverage then recovered and analyzed near-complete genome sequences from 8 linked patients. Processing higher number of samples per MiSeq run, and thus lowering the sequence coverage, would decrease the sequencing cost. We demonstrated that while *de novo* assembly may not be possible at lower level of coverage, reference guided assembly can be an effective alternative if a close reference exists. Sequence data obtained from this approach can be used to comprehensively analyze intra- and inter- host genetic variation and identify recombination events throughout the NoV genome.

## Ethics statement

A formal consent was not necessary because the study participants were anonymized.

## Availability of supporting data

The data sets supporting the results of this article are included within the article. The Illumina MiSeq short read sequences are deposited in the NCBI database.

## Author contributions

NN and JR designed and initiated the project. NN carried out all laboratory works including RNA extraction, quantification and RNA-Seq. NN and JR prepared the manuscript for publication. NP performed all the bioinformatics analysis. JR, SB, and NC supervised the project. All authors agreed with the final draft of the manuscript. All authors have read and approved the final manuscript.

### Conflict of interest statement

The authors declare that the research was conducted in the absence of any commercial or financial relationships that could be construed as a potential conflict of interest.

## Abbreviations

NGS: Next-generation Sequencing
NoV: Norovirus
ORF: Open reading frame
RdRp: RNA dependent RNA polymerase
WGS: Whole genome sequencing.
