# Supplementary material for: Characterization of the Genomic Diversity of Norovirus in Linked Patients Using a Metagenomic Deep Sequencing Approach
Source: Front Microbiol. 2017 Jan 31;8:73. doi: 10.3389/fmicb.2017.00073 (PMC5282449; doi:10.3389/fmicb.2017.00073)
Supplement: Supplementary file 1 [file Image1.pdf]

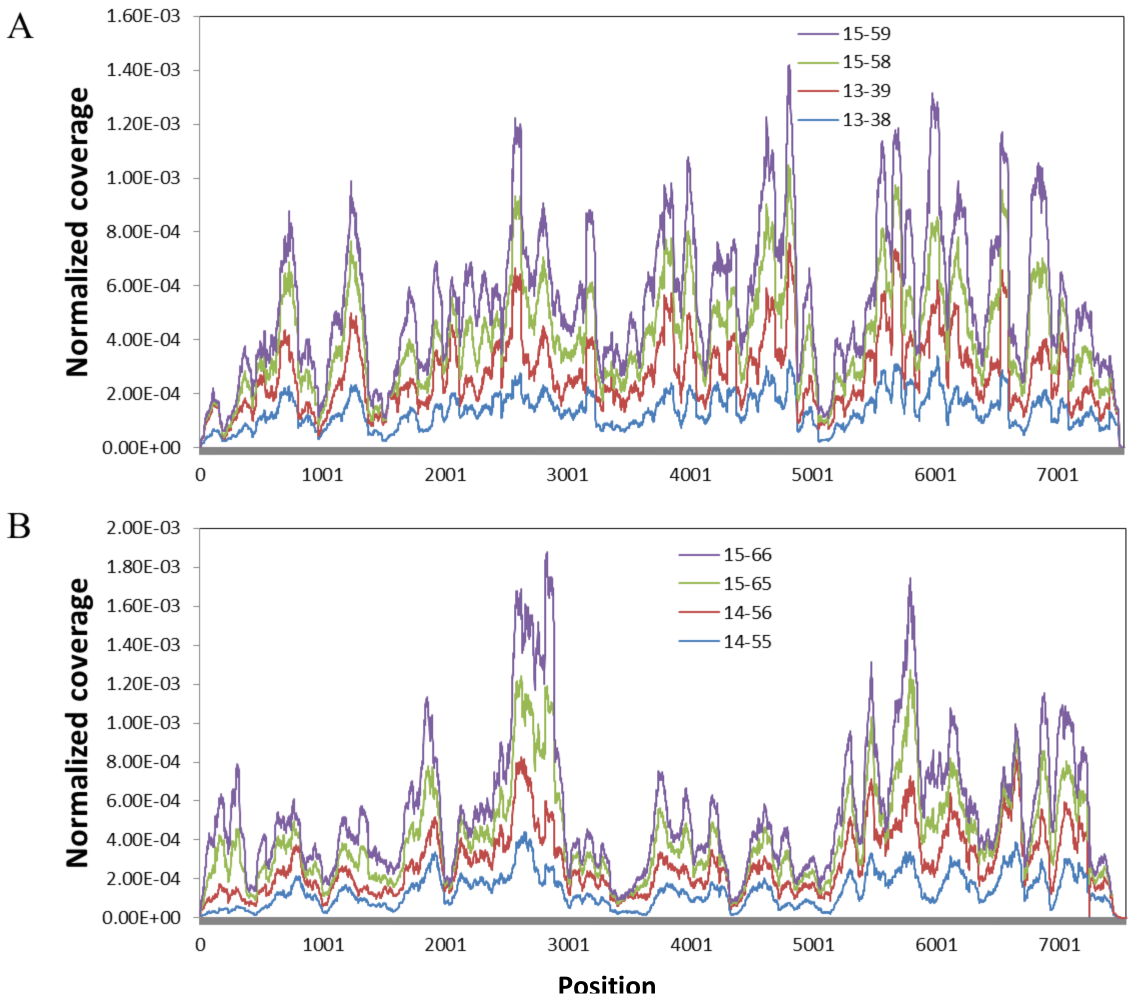

Supplementary Figure 1. Distribution of NoV reads across the sequenced genomes from GII.4 (A) and GII.6 (B). Coverage was calculated as the total number of reads covering a given nucleotide and was normalized by the sum of total coverage across the genome. i.e. at each residue, the coverage was divided by the total coverage and the sum of normalized coverage equals one.
